# Health-Related Quality of Life Among Food Bank Users in Spain: A Cross-Sectional Study

**DOI:** 10.3390/healthcare14091121

**Published:** 2026-04-22

**Authors:** Antonio Brugos-Larumbe, Alba Equiza-Vaquero, Carmen Hugo-Vizcardo, Laura Guillen-Aguinaga, Francisco Guillen-Grima, Ines Aguinaga-Ontoso

**Affiliations:** 1 Fundación Banco de Alimentos de Navarra, Polígono Comarca I (Agustinos), Calle Soto de Aizoáin 2-095, 31013 Pamplona, Spain; ablm649@gmail.com (A.B.-L.); trabajosocial@bancoalimentosnavarra.org (A.E.-V.); carmeny.hugov@pucp.pe (C.H.-V.); 2 Department of Nursing, Clinica Universidad de Navarra, 28027 Madrid, Spain; lguillen@alumni.unav.es; 3 Department of Preventive Medicine, Clinica Universidad de Navarra, 31008 Pamplona, Spain; 4 Department of Health Sciences, Public University of Navarra, 31008 Pamplona, Spain; 5 Group of Clinical Epidemiology, Area of Epidemiology and Public Health, Healthcare Research Institute of Navarre (IdiSNA), 31008 Pamplona, Spain; 6 CIBER in Epidemiology and Public Health (CIBERESP), Institute of Health Carlos III, 46980 Madrid, Spain

**Keywords:** health-related quality of life, food insecurity, EuroQol EQ-5D-5L, food banks, community epidemiology

## Abstract

**Highlights:**

**What are the main findings?**

- Users of the Navarra Food Bank showed impaired HRQoL, with a mean EQ-VAS of 73.56 and a mean EQ-5D-5L utility index of 0.815.
- Among Navarra Food Bank users, the most frequently affected EQ-5D-5L dimensions were anxiety/depression (62.9%) and pain/discomfort (55.7%).

**What are the implications of the main findings?**

- These findings indicate that HRQoL assessment may provide useful complementary information when evaluating socially vulnerable populations, especially where anxiety/depression and pain/discomfort are highly prevalent.
- They also underscore the need for future longitudinal, standardized comparative studies to better define the extent and determinants of HRQoL inequalities among food-insecure populations.

**Abstract:**

**Background**: Food bank users experience food insecurity, a social determinant of health linked to poorer physical and mental health. However, evidence on the health-related quality of life (HRQoL) of food bank users in Spain is scarce. **Objectives**: This study sought to assess HRQoL among users of the Navarra Food Bank and identify associated sociodemographic factors. **Methods**: We performed a cross-sectional study of heads of household using the Navarra Food Bank. A simple random sample of 350 participants was selected from a population of 2749 families. HRQoL was assessed by telephone using the EQ-5D-5L. We described the prevalence of problems in the five EQ-5D-5L dimensions, calculated the EQ-5D-5L utility index using the Spanish value set, and analyzed EuroQol Visual Analogue Scale (EQ-VAS) scores. Associations with sociodemographic characteristics were examined using multivariable general linear models. **Results**: Mean EQ-VAS was 73.56 (95% CI: 71.62–75.50), and mean EQ-5D-5L utility index was 0.815 (95% CI: 0.800–0.831). The most frequently reported problems were anxiety/depression (62.9%) and pain/discomfort (55.7%), while mobility (25.5%), usual activities (19.7%), and self-care (8.7%) were less commonly affected. Older age was significantly associated with both EQ-VAS and EQ-5D-5L utility index. Employment status and nationality were significantly associated with EQ-VAS, whereas sex was significantly associated with the EQ-5D-5L utility index. **Conclusions**: HRQoL was impaired among users of the Navarra Food Bank, with the greatest burden observed in the anxiety/depression and pain/discomfort dimensions. Older age and selected sociodemographic characteristics were associated with poorer HRQoL. Given the cross-sectional design, the findings should be interpreted as associative rather than causal.

## 1. Introduction

Food banks (FBs) are non-profit organizations that help alleviate hunger and poverty among socially vulnerable populations by providing immediate responses to food insecurity (FI), a phenomenon that persists even in high-income countries. Systematic reviews indicate that food banks can improve food security and diet quality among users, although they rarely eliminate FI [1,2,3].

In Spain and the wider Southern European context, food banks represent an important emergency response to FI. However, research in this setting has focused mainly on nutritional status and dietary intake, whereas North American studies have more extensively examined the broader impact of FI on health-related quality of life (HRQoL). Consequently, important evidence gaps remain regarding the HRQoL of food bank users in Southern Europe, where differences in social protection systems may shape how FI affects physical and mental health [4,5,6,7].

FI is a recognized social determinant of health and has been associated with poorer self-perceived health, mental disorders, chronic disease burden, greater healthcare use, and poorer quality of care in vulnerable populations [8,9,10,11,12]. HRQoL is commonly assessed using standardized generic instruments, among which the EQ-5D-5L is widely used in population health research [13,14,15,16].

Available evidence indicates that FI is associated with poorer HRQoL, but most studies have been conducted in North America. Research from the United States and Canada has consistently shown lower HRQoL among food-insecure individuals and food bank users, particularly in the mental domain, whereas evidence from Southern Europe remains scarce [17,18,19,20]. FI may impair HRQoL through multiple pathways, including chronic stress, material deprivation, and poorer physical and mental health [21,22,23,24].

The EQ-5D-5L is a widely used instrument for assessing HRQoL and is available in more than 170 languages [25]. It has been validated in the Spanish population [26,27] and has been used in national health surveys in Spain [28]. In Europe, evidence remains limited. In Portugal, a population-based study using the EQ-5D-3L found lower HRQoL in food-insecure households, whereas in the Netherlands, research on food bank users has focused mainly on nutritional vulnerability rather than HRQoL. Overall, studies of food bank users in Europe have concentrated more on diet and nutritional status than on standardized assessment of HRQoL [29,30].

In Spain, studies using the Spanish EQ-5D-5L value set have identified important inequalities in HRQoL associated with sociodemographic and health-related factors, supporting the relevance of this instrument for population health research [31].

At the regional level, the EQ-5D instruments have already been used in Navarra population studies, including the 2012 Navarra Situation Diagnosis and subsequent Navarra Social and Living Conditions Surveys [32,33]. Navarra is therefore a relevant setting for examining how FI affects HRQoL in a Spanish region characterized by relatively strong social protection policies.

Despite international evidence linking FI to poorer health outcomes and lower HRQoL, studies evaluating these outcomes among food bank users in Spain remain scarce. Against this background, this study aimed to assess HRQoL among users of the Navarra Food Bank and to examine its association with selected sociodemographic characteristics.

## 2. Materials and Methods

### 2.1. Study Design and Participants

A cross-sectional study was conducted based on a telephone survey of family heads who were users of the Navarra Food Bank, previously assessed by Social Services and certified as lacking financial resources. The study population consisted exclusively of current Navarra Food Bank users who had previously undergone a social services assessment for eligibility. The surveys were conducted between November 2024 and February 2025.

### 2.2. Sampling

The head of household (household reference person) was defined as the primary adult responsible for the household, including fathers or mothers in two-parent households, single parents, and individuals living alone. A sample size of 338 participants was calculated to estimate a population proportion, assuming a margin of error of 5%, a confidence level of 95%, and the most unfavorable scenario (*p* = 0.5; q = 0.5). The selection was made by simple random sampling from the list of Food Bank beneficiaries. Consequently, the source population does not include food-insecure individuals who do not seek or do not access food-bank assistance.

**Figure 1:**
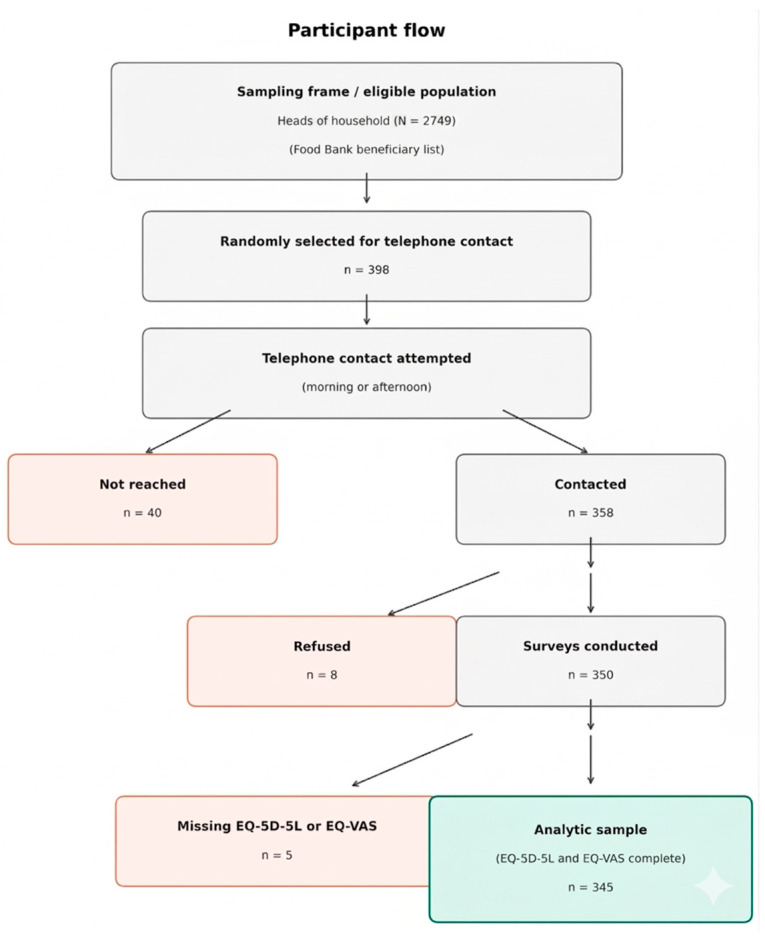
Participant flow diagram for sampling, recruitment, and survey completeness.

Initially, 398 individuals were selected and contacted by telephone in the morning or afternoon. Of these, 350 participants completed the survey, corresponding to a response rate of 87.9%. Five participants had missing responses on one or more EQ-5D-5L dimensions or on the EuroQol Visual Analogue Scale (EQ-VA)S and were excluded, yielding a final analytic sample of 345 participants with complete HRQoL data for all analyses (Figure 1). Sociodemographic characteristics of the fully recruited sample (N = 350) are presented in Table 1 and Table 2. Missing data patterns are detailed in Supplementary Table S1 (Figure 1).

### 2.3. Measuring Instrument

HRQoL was assessed using the EQ-5D-5L scale, in its Spanish-validated version. The questionnaire explores five dimensions: mobility, self-care, daily activities, pain/discomfort, and anxiety/depression. The EQ-VAS was also included, in which participants rated their current health status on a scale of 0 to 100, with 0 representing the worst possible health status and 100 the best.

The questionnaire also collected sociodemographic variables, including age, sex, country of origin (Spanish/foreign), educational level, employment status, housing characteristics, household size, and income-support variables.

### 2.4. Data Collection Procedure

Prior to the start of the fieldwork, 30 pilot interviews were conducted to standardize interview and assessment criteria. The interviews were conducted by telephone and in Spanish. When the interviewee was from Morocco and did not understand Spanish, the interview was conducted in Moroccan Arabic using the validated Moroccan Arabic version of the ED by two bilingual Arabic-speaking students, who were responsible for interviewing Arabic-speaking participants [34,35,36]. The interviews were conducted by telephone in Spanish or Arabic using a standardized protocol.

To ensure transparency and participant understanding, a formal script was followed to obtain verbal informed consent (the English translation of this script is provided in Supplementary File S1). The entire team received specific training in interview techniques and in the administration of the EQ-5D-5L scale, with special emphasis on the correct application of the EQ-VAS.

To ensure reliability and reproducibility [37], the research team underwent standardized training in telephone interviewing techniques and in administering the EQ-5D-5L scale, following established consortium guidelines for remote assessment.

### 2.5. Statistical Analysis

Categorical variables were described using frequencies and percentages, and comparisons of proportions were made using the chi-square test, calculating 95% confidence intervals. Numerical variables were described using descriptive statistics and compared using Student’s *t*-test or nonparametric tests (Mann–Whitney U or Kruskal–Wallis), depending on the distribution of the data. The level of statistical significance was set at *p* < 0.05.

For the EQ-5D-5L scale, the prevalence of problems in each of its five dimensions was estimated. EQ-VAS scores were also analyzed, and the EQ-5D-5L Index was calculated using the Spanish preference set, which assigns a numerical value to self-perceived health status.

Missing data were handled using complete-case analysis. Accordingly, both EQ-VAS and EQ-5D-5L utility index analyses were conducted on 345 complete cases. HRQoL was analyzed using the original continuous outcomes derived from the EQ-5D-5L instrument: the EQ-VAS score and the EQ-5D-5L utility index. First, descriptive analyses were conducted to summarize HRQoL by sociodemographic characteristics. Subsequently, multivariable general linear models were fitted separately for EQ-VAS and EQ-5D-5L utility index. In the main adjusted models, age was included as a continuous covariate, while sex, education level, nationality, and employment status were entered as fixed factors. These covariates were selected a priori based on their relevance to the study question and their availability in the dataset. Only main effects were included.

Effect sizes were expressed as partial eta squared. Homogeneity of variances was assessed using Levene’s test. Missing data were handled by complete-case analysis for each multivariable model. Statistical significance was set at *p* < 0.05. Multicollinearity was assessed using tolerance and variance inflation factor (VIF) values in linear regression models that included the same predictors as the main multivariable analyses. No relevant multicollinearity was identified, with VIF values close to 1 in both models. To assess the robustness of the findings, two sensitivity analyses were performed. First, household size was added to the main adjusted models. Second, an alternative socioeconomic model was fitted, including age and household size as continuous covariates, and sex, education level, nationality, housing type, Minimum Income Scheme (IMV), and Guaranteed Income/IMV as fixed factors.

As exact interview dates were not retained in the anonymized dataset, a sensitivity analysis was conducted using interview-order quartiles as a proxy for timing during fieldwork. Differences in EQ-VAS and EQ-5D-5L utility index across interview-order quartiles were examined using one-way ANOVA.

Statistical analyses were performed using IBM SPSS Statistics software, version 22.

### 2.6. Ethical Considerations

The study was approved by the Ethics, Animal Experimentation, and Biosafety Committee of the Public University of Navarra (UPNA) under code PI-021/24 on 28 October 2024. Informed consent was obtained from all subjects involved in the study. The procedure included a detailed explanation of data protection rights and the voluntary nature of participation, as documented in the verbal consent script (Supplementary File S1).

## 3. Results

### 3.1. Participation and Sample

The sample analyzed consisted of 350 users of the Navarra Food Bank, of whom 70.0% were women. The average age was 45.5 years (SD: 12.3), with no statistically significant differences between men and women (Table 1 and Table S1).

Most of the people surveyed were in the 30–44 and 45–59 age groups. In terms of educational level and employment status, basic levels of education and unemployment or precarious employment predominated. Table 2 provides a detailed overview of the main sociodemographic and socioeconomic characteristics of the population studied. Overall, the sample represents an adult population in a situation of socioeconomic vulnerability, with a high burden of family responsibilities and unstable working conditions, consistent with the profile of food bank users.

### 3.2. EQ-5D-5L Results (Dimensions)

The dimension with the highest prevalence of problems was anxiety/depression, followed by pain/discomfort. More than half of the respondents reported some degree of impairment in both dimensions. In contrast, the prevalence of problems in mobility, self-care, and daily activities was significantly lower. In all dimensions, anxiety/depression and pain/discomfort showed the highest prevalence of reported problems (Table 3).

### 3.3. EQ-VAS and EQ-5D-5L Utility Index According to Sociodemographic Characteristics

The mean EQ-VAS score was 73.56 points, with slightly higher values in men than in women. A clear age gradient was observed, with higher scores in younger groups and a progressive decline from the age of 45 onwards.

Across subgroups, EQ-VAS scores were slightly higher in men than in women and showed a clear age gradient, with lower values in older age groups (Table 4).

The EQ-5D-5L utility index showed a similar pattern, with lower values in women (Table 5).

#### Additional Age- and Sex-Standardized Analysis

As an additional descriptive analysis, we performed direct age- and sex-standardization of the study estimates (Supplementary Table S1). Standardized estimates were slightly lower than crude values for both EQ-VAS (71.60 vs. 73.56) and the EQ-5D-5L utility index (0.800 vs. 0.815).

### 3.4. Multivariable Linear Models of HRQoL Outcomes

To further examine the association between sociodemographic characteristics and HRQoL, multivariable general linear models were fitted separately for EQ-VAS and the EQ-5D-5L utility index. In the main adjusted models, age was included as a continuous covariate, while sex, education level, nationality, and employment status (recoded into three categories: employed, unemployed, and inactive) were entered as fixed factors. (Table 6) In the EQ-VAS model, the explanatory variables accounted for 15.3% of the total variance (adjusted R^2^ = 0.117). Older age was significantly and negatively associated with the self-perceived health score (F = 17.610; *p* < 0.001; eta^2^ *p* = 0.050). Similarly, the recoded employment status was found to be a significant determinant of EQ-VAS (F = 5.146; *p* = 0.006; eta^2^ *p* = 0.030). Nationality was also significantly associated with EQ-VAS (F = 4.545; *p* = 0.034; partial eta squared = 0.013). In contrast, sex (*p* = 0.060) and education level (*p* = 0.107) did not reach statistical significance in this model.

The EQ-5D-5L utility index model explained 13.9% of the variance (adjusted R^2^ = 0.102). In this analysis, age remained the strongest predictor (F = 18.351; *p* < 0.001; eta^2^ *p* = 0.052). Unlike the visual scale model, sex was significantly associated with the utility index (F = 5.194; *p* = 0.023; eta^2^ *p* = 0.015). While education level showed significance in initial main effects (F = 2.777; *p* = 0.041), the overall association in the final adjusted model was F = 2.328; *p* = 0.074. No statistically significant association was observed between the three-category employment status and the utility index (F = 1.281; *p* = 0.279).

Collinearity diagnostics indicated no problematic multicollinearity among predictors in either model. In the EQ-VAS model, tolerance values ranged from 0.978 to 0.991, and VIF values ranged from 1.009 to 1.023; in the EQ-5D-5L utility index model, tolerance values ranged from 0.976 to 0.991, and VIF values ranged from 1.009 to 1.025.

In sensitivity analyses, the main findings were broadly robust to further adjustment for additional socioeconomic vulnerability indicators. When household size was added to the main adjusted models, age remained significantly associated with both EQ-VAS and the EQ-5D-5L utility index; nationality remained associated with EQ-VAS, and sex remained associated with the EQ-5D-5L utility index, whereas household size was not significantly associated with either outcome. In an alternative socioeconomic model including housing type, Minimum Income Scheme (IMV), Guaranteed Income/IMV, and household size and age, age remained significantly associated with both HRQoL measures; nationality remained associated with EQ-VAS; and sex remained associated with the EQ-5D-5L utility index. Housing type, Minimum Income Scheme (IMV), Guaranteed Income/IMV, and household size were not significantly associated with either outcome (Supplementary Tables S2 and S3).

In a sensitivity analysis using interview-order quartiles as a proxy for fieldwork timing, EQ-VAS differed significantly across quartiles (F = 3.438, *p* = 0.017), although no linear trend was observed. In contrast, the EQ-5D-5L utility index did not differ significantly across interview-order quartiles (F = 1.408, *p* = 0.240).

## 4. Discussion

Health status is strongly influenced by non-medical factors, including educational level, access to adequate food, decent housing, and working conditions [8]. In this context, there is growing interest in examining the association between food insecurity and health outcomes [8,38,39], as well as identifying which HRQOL measures best reflect the experience of people who suffer from it.

The EQ-5D-5L is a widely validated instrument that has enabled the identification of significant differences with respect to the general population. Although it was designed for face-to-face administration, its use over the telephone has been widely employed and validated in previous studies [40,41].

In fact, the EuroQol consortium currently has specific guidelines for the telephone administration of the EQ-5D, confirming its validity and reliability in this format [42].

The HRQoL profile observed in this study was characterized by a high prevalence of problems in the anxiety/depression and pain/discomfort dimensions. In an additional descriptive analysis, direct age- and sex-standardization using the 2024 Navarra population as the reference yielded slightly lower estimates than the crude sample values (EQ-VAS: 71.60 vs. 73.56; EQ-5D-5L utility index: 0.800 vs. 0.815). For EQ-VAS, the standardized estimate remained below the available Navarra population reference value (88.1), suggesting that differences in age and sex distribution do not fully account for the observed gap. However, these comparisons should be interpreted cautiously, as they remain descriptive, and other differences between populations may also contribute to the observed contrast [33].

When age- and sex-standardized estimates were examined, the EQ-VAS value remained below the available Navarra population reference value (88.1), which provides descriptive support for the finding of poorer self-perceived health in this sample.

This overall pattern is in line with previous reports from other countries, including the United States and Canada, where food insecurity and food bank use have also been associated with poorer HRQoL and greater mental health burden. Nevertheless, these international comparisons should be interpreted with caution because the underlying populations, study designs, and outcome definitions differ across studies [17,43].

Our results show that poorer HRQoL was particularly associated with emotional distress, especially in the anxiety/depression dimension, a pattern consistent with life insecurity, chronic stress, and economic uncertainty described in previous literature. These findings are consistent with studies conducted in Spain [44], which described an increase in diagnoses of depression and anxiety disorders in the most socioeconomically disadvantaged groups following the 2008 economic crisis [44]. Similarly, a recent systematic review across 24 countries confirms the association between economic hardship and deterioration in mental health [45].

Overall, our data show that emotional distress is more prevalent than severe physical impairment, consistent with the framework of social determinants of health [22].

The multivariable models showed a differentiated pattern of association: age was consistently associated with both HRQoL outcomes, whereas employment status was associated only with EQ-VAS. Age was a robust predictor in both models, indicating that, even within a socioeconomically vulnerable group, the natural decline in health associated with aging significantly compounds the burden of poverty.

The lack of a clear independent association with education level may reflect the heterogeneity of this population, in which educational qualifications do not necessarily translate into employment opportunities or social resources, particularly among foreign-born participants. A key finding is the divergence in how employment status (recoded into three categories: employed, unemployed, and inactive) impacts the two HRQoL measures. While employment status was a highly significant predictor of the global EQ-VAS score (*p* = 0.006), it did not reach statistical significance for the EQ-5D-5L utility index (*p* = 0.279).

This discrepancy suggests that being unemployed or inactive in a context of high vulnerability carries a heavy “holistic” psychological and social weight that users capture more accurately on a 0–100 scale. In contrast, the utility index, which is weighted based on specific physical and mental dimensions, may be less sensitive to the overall existential stress of joblessness if it has not yet manifested as a severe functional limitation.

Furthermore, the significant association of sex with the utility index (*p* = 0.023), but not with the EQ-VAS (*p* = 0.060), aligns with our descriptive findings. Women in this cohort reported a higher prevalence of problems in specific dimensions such as pain/discomfort and anxiety/depression, which are heavily weighted in the Spanish value set for the EQ-5D-5L index. This pattern indicates that, while both genders experience a similar global health malaise (as reflected in the VAS), women experience more localized and dimension-specific impairments, which lower their calculated utility index.

Finally, the significance of nationality in the EQ-VAS model (*p* = 0.034) indicates that cultural or migratory factors may also play a role in how individuals evaluate their overall health status. These results underscore the complexity of social determinants; once a state of severe financial resource deficiency is established, health challenges are driven by a combination of aging, gender-specific vulnerabilities, and the chronic stress of labor instability. These results are consistent with previous evidence indicating that psychological well-being and limitations in daily activities often carry the greatest explanatory weight in HRQoL models once a state of vulnerability is established [46,47].

Among the strengths of the study are an adequate sample size, the use of a validated instrument such as the EQ-5D-5L, and the inclusion of the Arabic-speaking population, which is usually underrepresented in this type of research.

However, the study has limitations inherent to its cross-sectional design, which preclude establishing causal relationships between receiving food aid and HRQoL—a distinction noted in previous longitudinal studies [48]. Residual confounding cannot be excluded. In particular, the duration of food insecurity was not collected in this study and therefore could not be incorporated into the adjusted analyses. Nevertheless, additional sensitivity analyses were performed using the socioeconomic vulnerability indicators available in the dataset, including household size, housing type, and income-support variables.

Beyond this design-related constraint, the timing of data collection (November to February) warrants careful consideration. This period coincides with winter in northern Spain, which may exacerbate self-reported pain and discomfort due to colder temperatures [49,50,51,52]. Additionally, the financial and social pressures associated with the end-of-year holiday season may have temporarily inflated the prevalence of anxiety and depression among these vulnerable households, who often struggle to meet the social expectations and expenses of this period [53,54,55].

Although exact interview dates were not retained in the anonymized dataset, a sensitivity analysis using interview-order quartiles as a proxy for timing during fieldwork suggested some non-linear variation in EQ-VAS. In contrast, no significant variation was observed for the EQ-5D-5L utility index. These findings do not allow direct assessment of month-specific or holiday-related effects, but suggest that any temporal variation during fieldwork was limited and inconsistent across HRQoL measures.

A potential selection bias must also be acknowledged [56,57]. This study included only current users of the Navarra Food Bank who had previously received social services; therefore, the findings are primarily generalizable to food bank users who actively seek assistance rather than to all individuals experiencing food insecurity. However, some food-insecure individuals may not seek help from social services or food banks because of shame, stigma, lack of information, or other psychosocial barriers. As a result, the study may underrepresent a subgroup of food-insecure people whose health and social profiles could differ from those of service users.

In addition, non-response bias cannot be excluded, as no comparative information was available for participants who did not complete the survey. Moreover, as the study relies exclusively on self-perceived health, the results may be subject to subjective bias [58,59,60,61]. Lastly, the absence of a matched comparison with the general population limits the ability to quantify the health gap attributable to food insecurity accurately. Future research should aim to compare food bank beneficiaries with food-insecure non-users to isolate better differences in HRQoL related to aid-program use.

Furthermore, the comparison with the general population of Navarra remains descriptive. Although we performed an additional age- and sex-standardized analysis, the comparison was not based on matched individual-level data, and other differences between populations may still contribute to the observed gap. Future studies should employ matched or longitudinal designs to provide a more refined quantification of HRQoL inequalities related to food insecurity.

## 5. Conclusions

This study provides novel evidence of impaired HRQoL among users of the Navarra Food Bank, with the greatest burden observed in the anxiety/depression and pain/discomfort dimensions. Older age and selected sociodemographic characteristics were associated with poorer HRQoL. Comparisons with the general population should be interpreted cautiously, even after age- and sex-standardization, because they remain descriptive.

## Figures and Tables

**Table 1:** Distribution of the sample by age and sex (N = 350).

Sex	N	Mean	SD
Male	105	47.2	13.2
Female	245	44.7	11.8
Total	350	45.5	12.3

**Table 2:** Main sociodemographic and socioeconomic characteristics of the study population.

Sociodemographic and Socioeconomic Characteristics	Categories	n (N = 350)	%
Employment Status	Full-time employee	21	6.0
	Part-time employee	70	20.0
	Unemployed	92	26.3
	Student	3	0.9
	Retired/Pensioner	22	6.3
	Self-employed	4	1.1
	Not working, not looking for work	59	16.9
	Not working due to disability/illness	20	5.7
	Lacks documentation	59	16.9
How many people live at home?	1	30	8.6
	2	56	16
	3	63	18
	4	72	20.6
	5	61	17.4
	≥6	68	19.4
Age Group	18 to 29 years old	36	10.3
	30 to 44 years old	143	40.9
	45 to 59 years old	123	35.1
	60 years or older	48	13.7
Nationality	Spain	74	21.1
	Other	276	78.9
Geographic Origin	Africa	93	26.6
	Asia	2	0.6
	Europe	107	30.6
	Latin America	148	42.3
Education Level	Unable to read or write	12	3.4
	No formal education (can read/write)	37	10.6
	Primary or secondary education	217	62
	University or vocational training	84	24

**Table 3:** EQ-VAS scores and prevalence of reported problems by EQ-5D-5L dimension and sex (n = 345).

	EQ-VAS Mean (95% CI)	Mobility (95% CI)	Self-Care (95% CI)	Daily Activities (95% CI)	Pain/Discomfort (95% CI)	Anxiety/Depression (95% CI)
Total	73.6 (71.6–75.5)	25.2% (20.9–30.1)	8.4% (5.7–11.7)	19.4% (15.5–23.9)	55.7% (50.4–60.9)	62.9% (57.8–68.0)
Male	75.8 (72.3–79.3)	24.5% (16.2–33.3)	8.8% (3.3–14.6)	19.8% (11.9–27.7)	45.1% (34.7–54.4)	56.9% (46.6–66.3)
Female	72.6 (70.2–74.9)	25.5% (20.3–31.3)	8.2% (5.1–12.2)	19.3% (14.6–24.7)	60.1% (54.1–66.4)	65.4% (59.6–71.6)

EQ-VAS = EuroQol Visual Analogue Scale.

**Table 4:** Descriptive statistics of EQ-VAS scores according to sociodemographic characteristics (n = 345).

	N	Mean 95% IC	SD	Median
Total	345	73.56 (71.62–75.50)	18.28	75.0
Sex				
Men	102	75.83 (72.35–79.36)	17.86	80.0
Women	243	72.60 (70.27–74.92)	18.41	75.0
Age groups				
18 to 29 years old	36	76.14 (69.64–82.64)	19.20	80.0
30 to 44 years old	142	77.99 (75.46–80.53) *	15.27	80.0
45 to 59 years old	121	70.72 (67.31–74.12) *	18.91	70.0
60 years old or older	46	65.33 (59.20–71.45) *	20.61	70.0
Nationality				
Spain	73	68.63 (63.43–73.83)	22.30	70.0
Other	272	74.88 (72.87–76.89)	16.85	80.0
Level of education				
Cannot read or write	11	72.73 (59.86–85.59)	19.15	70.0
No formal education, but can read and write	36	73.89 (67.39–80.39)	19.20	80.0
Primary or secondary education	214	75.01 (72.64–77.39)	17.65	80.0
University education or vocational training	84	69.82 (65.67–73.98)	19.14	70.0

* ANOVA with Scheffé test: significant differences (<0.05) between the 60 or older group and the 18–29 and 30–44 age groups, and the 30–44 age group with the 45–59 age group.

**Table 5:** EQ-5D-5L utility index by sex (n = 345).

Variable	Total (n = 345)	Male (n = 104)	Female (n = 242)
EQ-5D-5L utility index *	0.815 (0.800–0.831)	0.834 (0.813–0.862)	0.806 (0.786–0.825)

* The EQ-5D-5L utility index was calculated using the Spanish population value set [26].

**Table 6:** Multivariable general linear models for EQ-VAS and EQ-5D-5L utility index in the main adjusted analysis (n = 345).

Outcome	Predictor	df	F	*p*-Value	Partial Eta Squared
EQ-VAS	Employment status	2	5.146	0.006	0.030
	Age	1	17.61	<0.001	0.050
	Sex	1	3.549	0.060	0.010
	Nationality	1	4.545	0.034	0.013
	Education level	3	2.049	0.107	0.018
EQ-5D-5L utility index	Employment status	2	1.281	0.279	0.008
	Age	1	18.351	<0.001	0.052
	Sex	1	5.194	0.023	0.015
	Nationality	1	0.633	0.427	0.002
	Education level	3	2.328	0.074	0.020

## Data Availability

The raw data supporting the conclusions of this article will be made available by the authors on request.

## Supplementary Materials

The following supporting information can be downloaded at https://www.mdpi.com/article/10.3390/healthcare14091121/s1: Table S1: Crude and age- and sex-standardized HRQoL estimates in the Navarra Food Bank sample; Table S2: Sensitivity analysis of multivariable models for HRQoL outcomes after additional adjustment for household size; Table S3: Sensitivity analysis of multivariable models for HRQoL outcomes after additional adjustment for housing and socioeconomic vulnerability indicators; Supplementary File S1: English translation of the verbal informed consent script.

## Abbreviations

The following abbreviations are used in this manuscript:

FB	Food Banks
FI	Food Insecurity
HRQoL	Health-Related Quality of Life
EQ-VAS	EuroQol Visual Analogue Scale
