# Supplementary material for: Health-Related Quality of Life Among Food Bank Users in Spain: A Cross-Sectional Study
Source: Healthcare (Basel). 2026 Apr 22;14(9):1121. doi: 10.3390/healthcare14091121 (PMC13163688; doi:10.3390/healthcare14091121)
Supplement: Supplementary file 1 [file healthcare-14-01121-s001.zip › SUPLEMENTARY FILE S1.pdf]

SUPLEMENTARY FILE S1

INFORMED CONSENT

- "Good morning, we are calling from the **Navarra Food Bank Foundation** (*Fundación Banco de Alimentos de Navarra*); my name is ………...
- We are conducting a study in collaboration with the **Public University of Navarra** with the aim of assessing your level of satisfaction with the services we provide and, simultaneously, gaining a better understanding of the sociodemographic characteristics and health-related quality of life of our beneficiaries.
- This information is essential for improving the care and resources we offer.
- Please be advised that the call may be recorded to comply with current data protection regulations.
- We inform you that your personal data will be processed solely for the purpose of conducting this study and will not be used for any other purposes under any circumstances.
- The processing of your data is only possible if you provide your explicit consent, in accordance with the provisions of data protection regulations.
- Your data will be retained for the period necessary to carry out this study, after which it will be deleted.
- I want to assure you that all information you provide will be treated with absolute confidentiality, in compliance with data protection regulations.
- Your responses will be anonymous and used solely for statistical purposes; therefore, you cannot be individually identified under any circumstances.
- Before we begin, I would like to confirm your express consent for the processing of your personal data.
- Participation is entirely voluntary, and you may choose not to answer any question or terminate the interview at any time if you wish.
- Participants waive any rights of an economic nature arising from their participation in the study.
- You may exercise your data protection rights at any time by contacting the BAN at 948 30 38 16 or via email at: ban@bancoalimentosnavarra.org.
- You also have the right to file a claim with the Spanish Data Protection Agency.
- If you wish to read the full Participant Information Sheet and additional information regarding data protection, you may request it, and we will provide it to you via email.
- The survey is brief and may last between 10 and 12 minutes.
- Do you declare that you have been informed about the study and agree to proceed with the interview?
- **Yes [ ] No [ ]**"

Original Spanish Version of the Informed Consent Script

CONSENTIMIENTO INFORMADO LEIDO AL PRINCIPIO DE LA LLAMADA TELEFONICA Y GRABADO

“Buenos días, le llamamos de Fundación Banco de Alimentos de Navarra soy ……….

Estamos realizando un estudio en colaboración con la Universidad Pública de Navarra con el objetivo de conocer su nivel de satisfacción con los servicios que ofrecemos y, al mismo tiempo, entender mejor las características sociodemográficas y calidad de vida relacionada con la salud de nuestros beneficiarios. Esta información es fundamental para poder mejorar la atención y los recursos que brindamos.

Le informamos de que la llamada podrá ser grabada para cumplir con la normativa vigente en materia de protección de datos.

Le informamos de que sus datos personales serán tratados con el único fin de llevar a cabo el estudio, no siendo usados, en ningún caso, para otras finalidades. El tratamiento de sus datos solo será posible si usted nos otorga su consentimiento explícito, de acuerdo con lo establecido en la normativa de protección de datos. Sus datos se conservarán durante el tiempo necesario para llevar a cabo el presente estudio, momento tras el cual serán suprimidos.

Quiero asegurarle que toda la información que nos proporcione será tratada con absoluta confidencialidad, cumpliendo con normativa de protección de datos. Sus respuestas serán anónimas y se utilizarán únicamente con fines estadísticos, por lo que en ningún caso se le podrá identificar de manera individual.

Antes de comenzar, quisiera confirmar su consentimiento expreso para llevar a cabo el tratamiento de sus datos personales. La participación es completamente voluntaria, y puede decidir no responder a alguna pregunta o finalizar la entrevista en cualquier momento si así lo desea. Los participantes renuncian a cualquier derecho de naturaleza económica por su participación en el estudio. Usted puede ejercitar sus derechos de protección de datos en cualquier momento dirigiéndose al BAN en el teléfono 948 30 38 16 o en el correo electrónico: ban@bancoalimentosnavarra.org

También tiene derecho a interponer una reclamación ante la Agencia Española de Protección de Datos.

Si desea leer la Hoja de Información al Participante completa y la información adicional en materia de protección de datos, nos la puede solicitar y se la facilitaremos por correo electrónico.

La encuesta es breve y puede durar entre 10 y 12 minutos.

¿Declara haber sido informado acerca del estudio y está de acuerdo en continuar con la entrevista?

Sí [ ]   **No [ ]** `
