# Supplementary material for: Health-Related Quality of Life Among Food Bank Users in Spain: A Cross-Sectional Study
Source: Healthcare (Basel). 2026 Apr 22;14(9):1121. doi: 10.3390/healthcare14091121 (PMC13163688; doi:10.3390/healthcare14091121)
Supplement: Supplementary file 1 [file healthcare-14-01121-s001.zip › Supplementary Table S1.pdf]

Supplementary Table S1. Crude and age- and sex-standardized HRQoL estimates in the Navarra Food Bank sample

Outcome	Crude mean (SD)	Age- and sex-standardized mean	Navarra reference value
EQ-VAS	73.56 (18.28)	71.60	88.1
EQ-5D-5L utility index	0.815 (0.146)	0.800	Not available

HRQoL=health-related quality of life; EQ-VAS=EuroQol visual analogue scale.

* Age- and sex-standardized estimates were calculated by direct standardization using the 2024 Navarra population as the reference population. Navarra reference values are shown for descriptive comparison only. Age-sex specific mean values for EQ-5D-5L utility index and EQ-VAS were first estimated in the Navarra Food Bank sample and then weighted according to the corresponding population proportions in Navarra.
