# Supplementary material for: Health-Related Quality of Life Among Food Bank Users in Spain: A Cross-Sectional Study
Source: Healthcare (Basel). 2026 Apr 22;14(9):1121. doi: 10.3390/healthcare14091121 (PMC13163688; doi:10.3390/healthcare14091121)
Supplement: Supplementary file 1 [file healthcare-14-01121-s001.zip › Supplementary Table S2.pdf]

Supplementary Table S2. Sensitivity analysis of multivariable models for HRQoL outcomes after additional adjustment for household size (n=345)

Outcome	Predictor	df	F	p-value	Partial eta squared	R^2	Adjusted R^2	n
**EQ-VAS**	Employment status	2	4.977	0.007	0.029	0.136	0.113	345
	Age	1	15.756	<0.001	0.045	0.136	0.113	345
	Sex	1	3.343	0.068	0.010	0.136	0.113	345
	Nationality	1	3.942	0.048	0.012	0.136	0.113	345
	Education level	3	1.976	0.117	0.017	0.136	0.113	345
	Household size	1	0.202	0.653	0.001	0.136	0.113	345
**EQ-5D-5L utility index**	Employment status	2	1.071	0.344	0.006	0.110	0.086	345
	Age	1	14.553	<0.001	0.042	0.110	0.086	345
	Sex	1	4.459	0.035	0.013	0.110	0.086	345
	Nationality	1	0.199	0.656	0.001	0.110	0.086	345
	Education level	3	2.190	0.089	0.019	0.110	0.086	345
	Household size	1	2.382	0.124	0.007	0.110	0.086	345

Footnote: General linear models with main effects only. Age and household size were included as continuous covariates; sex, nationality, education level, and employment status were entered as fixed factors. Effect sizes are presented as partial eta squared. These models correspond to the main adjusted model plus household size.
