# Supplementary material for: Health-Related Quality of Life Among Food Bank Users in Spain: A Cross-Sectional Study
Source: Healthcare (Basel). 2026 Apr 22;14(9):1121. doi: 10.3390/healthcare14091121 (PMC13163688; doi:10.3390/healthcare14091121)
Supplement: Supplementary file 1 [file healthcare-14-01121-s001.zip › Supplementary Table S3.pdf]

Supplementary Table S3. Sensitivity analysis of multivariable models for HRQoL outcomes after additional adjustment for housing and socioeconomic vulnerability indicators

Outcome	Predictor	df	F	p-value	Partial eta squared	R^2	Adjusted R^2	n
**EQ-VAS**	Education level	3	1.407	0.240	0.013	0.122	0.093	345
	Housing type	2	1.362	0.258	0.008	0.122	0.093	345
	Sex	1	3.849	0.051	0.011	0.122	0.093	345
	Nationality	1	4.849	0.028	0.014	0.122	0.093	345
	Minimum income benefit (IMV)	1	0.955	0.329	0.003	0.122	0.093	345
	Rent support / IMV	1	1.543	0.215	0.005	0.122	0.093	345
	Household size	1	0.968	0.326	0.003	0.122	0.093	345
	Age	1	21.522	<0.001	0.061	0.122	0.093	345
**EQ-5D-5L utility index**	Education level	3	2.309	0.076	0.020	0.111	0.082	346
	Housing type	2	0.043	0.958	0.000	0.111	0.082	346
	Sex	1	4.549	0.034	0.013	0.111	0.082	346
	Nationality	1	0.582	0.446	0.002	0.111	0.082	346
	Minimum income benefit (IMV)	1	2.187	0.140	0.007	0.111	0.082	346
	Rent support / IMV	1	0.003	0.956	0.000	0.111	0.082	346
	Household size	1	2.976	0.085	0.009	0.111	0.082	346
	Age	1	17.412	<0.001	0.050	0.111	0.082	346

Footnote: General linear models with main effects only. Age and household size were included as continuous covariates; education level, housing type, sex, nationality, minimum income benefit, and rent support/IMV were entered as fixed factors. EQ-VAS was analysed in 345 complete cases and EQ-5D-5L utility index in 346 complete cases. Effect sizes are presented as partial eta squared. These models represent an alternative socioeconomic sensitivity analysis incorporating housing instability and income-support indicators available in the dataset
